# Revisiting extended-criteria donors in lung transplantation

**DOI:** 10.1016/j.jhlto.2026.100689

**Published:** 2026-09-18

**Authors:** Cedric Vanluyten, René Novysedlak, Annalisa Barbarossa, An-Lies Provoost, Jan Van Slambrouck, Lori van Roozendaal, Maxime Van den Brande, Andrea Zajacova, Bart M. Vanaudenaerde, Robin Vos, Robert Lischke, Dirk E. Van Raemdonck, Laurens J. Ceulemans

**Affiliations:** a Department of Thoracic Surgery, University Hospitals Leuven, Leuven, Belgium; b Department of Chronic Diseases and Metabolism, Laboratory of Respiratory Diseases and Thoracic Surgery (BREATHE), KU Leuven, Leuven, Belgium; c Prague Lung Transplant Program, 3rd Department of Surgery, First Faculty of Medicine, Charles University, Motol and Homolka University Hospital, Prague, Czech Republic; d Department of Pulmonology, University Hospitals Leuven, Leuven, Belgium

**Keywords:** age, donation after circulatory death, extended-criteria donor, lung transplantation, smoking

## Abstract

Lung transplantation (LTx) is an established life-saving therapy for patients with end-stage lung disease. Despite continuous improvements in surgical technique, immunosuppressive regimen and perioperative care, organ shortage remains the principal limitation to broader access. Historically, conservative donor criteria have contributed to the underutilization of potentially transplantable lungs. Over time, lungs from donors outside standard criteria – commonly referred to as extended-criteria donors (ECD) – have been increasingly accepted. This narrative review evaluates contemporary evidence on the experience and outcomes of specific ECD lungs in LTx. We focus first on typically defined ECD including older donor age, smoking history and substance abuse, oxygenation status, radiological abnormalities, microbial colonization, donation after circulatory death, and extended ischemic times. Subsequently, we look at specific donor traits tempering them as emerging ECD. Finally, we evaluate the role and potential benefits of novel preservation and assessment techniques such as controlled hypothermic storage and ex vivo lung perfusion. While accumulating data support the safe use of ECD lungs under appropriate conditions, a paradigm shift toward individualized donor assessment rather than rigid thresholds is mandatory to sustainably expand the donor pool and reduce waitlist mortality.

## Introduction

Lung transplantation (LTx) has evolved from an experimental procedure into an established therapeutic option for carefully selected patients with end-stage lung disease.^1^ Progress in surgical techniques, immunosuppressive regimens, antimicrobial prophylaxis, and perioperative care have significantly improved patient outcomes. Despite these advances, access to transplantation remains limited by a persistent shortage of suitable donor organs, which continues to be a major unresolved challenge in the field. Historically, donor selection relied on standard criteria that were largely based on empirical clinical experience around the 1990s.^2^, ^3^ These included donor age <50 years (initially <45 years), absence of smoking history and substance abuse, PaO₂/FiO₂ > 350 mmHg, clear chest radiograph, absence of microbial colonization, ischemic time <6 h, and donation after brain death (DBD). While these stringent criteria aimed to optimize post-transplant outcomes, they also excluded a substantial number of potentially usable donor lungs.

Nevertheless, as waitlist mortality remained unacceptably high and donor characteristics evolved over time, these traditional criteria were increasingly challenged for being overly restrictive. Supported by the International Society for Heart and Lung Transplantation (ISHLT), the use of extended-criteria donors (ECD) has progressively expanded.^4^ Data from the United Network for Organ Sharing (UNOS) illustrate this shift, with a decrease of standard criteria lung donors from 34% in 2005 to only 14% in 2018.^5^ In parallel, successful LTx has increasingly been reported for specific donor comorbidities that were previously considered unsuitable for transplantation (including viral infections, asthma and malignancies), positioning them as emerging ECD. For this narrative review, we performed targeted PubMed searches between focusing on ECD in LTx. Here, we critically evaluate the role of ECD lungs in LTx, with particular focus on clinical outcomes, current limitations and implications for future donor selection strategies.

## Defining extended-criteria donors for lung transplantation

The definition of lungs obtained from ECD donors varies across centers and geographic regions, reflecting differences in clinical practice, resource availability, and risk tolerance. The lungs’ particular vulnerability to infection, trauma, ventilator-associated damage, and ischemia–reperfusion injury, partly explains why donor selection criteria have historically been more restrictive than those for other solid organs. ECD lungs are defined as donor lungs that deviate from standard criteria, and involve donor lungs ≥50 years, donors with smoking history or substance abuse, suboptimal oxygenation, traumatic injuries leading to abnormal imaging findings, microbial colonization, and longer ischemia time (Figure 1). Although donation after circulatory death (DCD) was historically considered an extended criterion, it now accounts for up to 50% of the donor pool in both European and North-American centers, thereby questioning its classification as an extended criterion.^6^ Nevertheless, the expansion of DCD heart transplantation and the use of uncontrolled lung donation (DCD-I or DCD-II), introduce new challenges. These developments may justify classifying specific DCD categories as an extended criteria.

**Figure 1:**
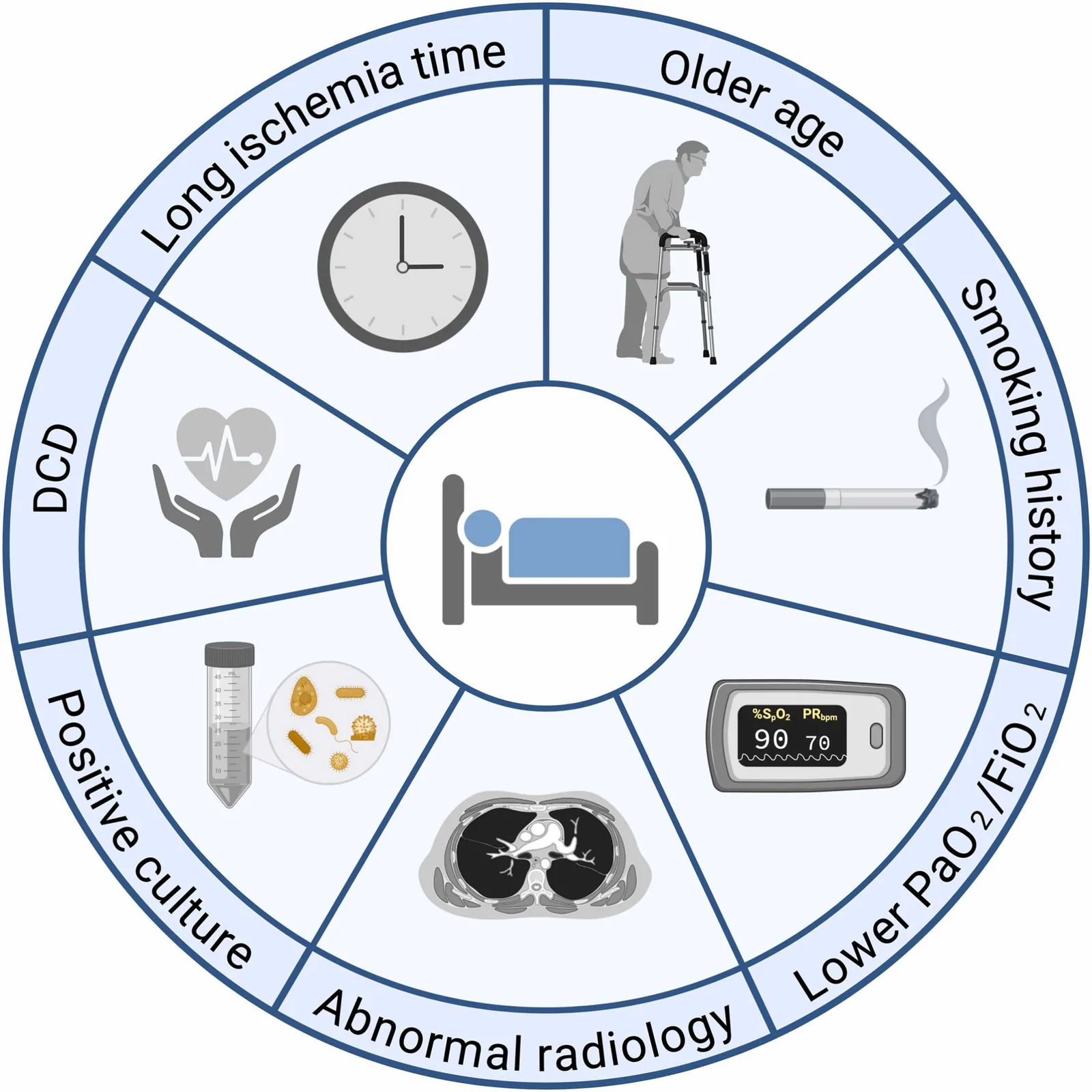
Overview of extended-criteria donors in lung transplantation.

Unlike other solid organs, however, donor lungs lack specific biomarkers, and pre-procurement assessment relies on a relatively limited set of functional and anatomical parameters, including oxygenation, chest radiographic and bronchoscopic findings. Although these parameters provide valuable information, they largely represent indirect surrogates of pulmonary function and do not adequately characterize regional ventilation-perfusion matching. This limitation is particularly relevant when assessing focal abnormalities, as currently available diagnostic tools often cannot reliably determine the functional contribution of individual lobes or distinguish reversible from irreversible injury. Macroscopic evaluation at the time of procurement, with the chest fully opened and both lungs ventilated, can provide an idea of the compliance (on inflation) and elasticity (on deflation) of the lungs. Also ex-vivo lung perfusion (EVLP) has emerged as a platform to perform functional assessment in case of doubt. Consequently, traveling to the donor hospital for in-situ inspection allows more lungs to be accepted than would be judged acceptable from the donor file alone.^7^

Furthermore, the presence of a single extended criterion does not necessarily imply inferior graft quality, whereas the cumulative burden of multiple risk factors appears to be more clinically relevant. To estimate donor lung acceptability, several scoring systems have been developed, including the Oto score, the Eurotransplant Lung Donor Score (ELDS), and the Lung Donor (LUNDON) score.^8^, ^9^, ^10^, ^11^ These models move beyond the binary classification of standard *versus* extended criteria by integrating multiple donor-related risk factors into a composite score. More recently, models incorporating both donor and recipient characteristics have demonstrated improved predictive performance, highlighting the intrinsic interplay between donor quality and recipient condition.^12^ Recipient-related factors, including the high urgency of transplantation due to the need for mechanical ventilation or extracorporeal membrane oxygenation (ECMO), significantly influence post-transplant outcomes and should therefore be carefully balanced against donor availability. Nevertheless, no currently available scoring system can fully capture the complexity of donor-recipient matching or reliably predict long-term outcomes in all cases. Consequently, contemporary donor assessment is increasingly shifting toward a more holistic evaluation of the donor lung’s overall functional and biological condition rather than relying on isolated variables (Figure 2). This shift reflects increasing confidence in the safe use of ECD, supported by advances in donor management, recipient selection, and perioperative care.

**Figure 2:**
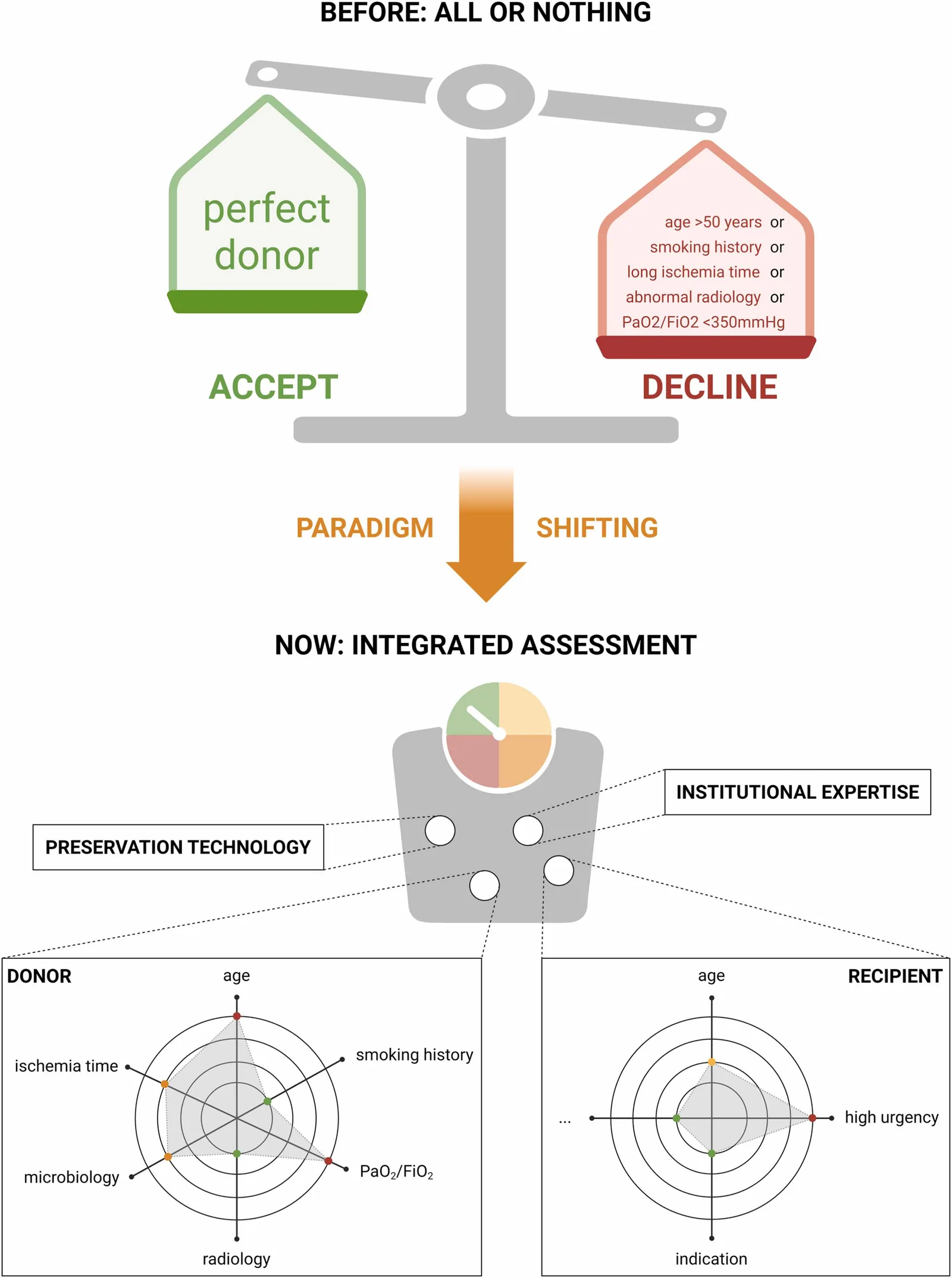
Evolution of lung donor assessment over time. Today, donor lung assessment integrates the recipients’ risk profile with donor characteristics, including precise radiological assessment and by improved preservation technologies.

## Increase of extended-criteria donors and clinical outcomes

### Donor age ≥ 50 years

As global life expectancy continues to rise, it is estimated that by 2050 more than 2 billion people will be older than 60 years.^13^ Avoiding a strict upper age limit for organ donation will, ceteris paribus, increase the overall availability of transplantable organs. However, due to age-related structural and immunologic changes, advanced donor age beyond 50 years has traditionally been viewed as a major risk factor for adverse outcomes after LTx. Furthermore, cellular aging – or senescence – promotes the secretion of pro-inflammatory cytokines and growth factors, which contributes to chronic low-grade inflammation or “inflammaging.” While other solid organ transplant programs were early to increase their age limit, LTx remained comparatively conservative regarding older donor lungs.^14^, ^15^ Particularly in liver transplantation, the boundaries of donor age continue to be challenged, as exemplified by the successful transplantation of a centenarian liver by the team of Pisa, Italy.^16^

Over the years, however, large cohort studies have demonstrated that donor age is not an independent predictor of post-transplant mortality or chronic lung allograft dysfunction (CLAD) development after LTx. In a propensity-matched analysis, our center demonstrated that septuagenarian donors provide comparable outcomes compared to donors <70 years regarding primary graft dysfunction (PGD), CLAD, and overall survival.^17^ Likewise, there was no difference in airway complications and postoperative lung function between donors <70 years and >70 years. Meanwhile, several centers have successfully transplanted lungs from donors aged ≥70 years and even LTx with donors ≥80 years have shown good short- and long-term outcomes.^18^, ^19^, ^20^, ^21^ In a multicenter analysis, Pradère et al. further demonstrated that lungs from older donors can be safely transplanted even into younger recipients, challenging the traditional “old-for-old” allocation paradigm.^22^ This concept was originally pioneered in kidney transplantation through the Eurotransplant Senior Program, allocating kidneys from donors >65 years to age-matched recipients.^23^

A recent case study reported successful bilateral LTx using lungs from a 94-year-old donor, highlighting the importance of biological rather than chronological age in donor assessment.^24^ This concept is further supported by the development of epigenetic aging clocks, which demonstrate that aging is a heterogeneous and measurable biological process. While some protective factors (e.g. exercise, caloric restriction, …) can decelerate the biological aging process, inflammation and comorbidities (such as chronic kidney disease) can accelerate aging.^25^ Taken together, these data suggest that donor age beyond 50 years should not be considered an absolute exclusion criterion, but rather as one component of a broader composite risk profile. Especially for older donors, strict assessment and balancing all donor characteristics towards the recipient profile remains essential. Future integration of biomarkers reflecting biological rather than chronological age may further refine donor selection and optimize the use of older donors in LTx.

### Smoking and substance abuse

Although global smoking prevalence dropped from 33% in 2000 to less than 20% in 2024, a history of tobacco use and active smoking remain common donor characteristics and reasons for lung refusal.^26^, ^27^ Smoking-related lung injury raises concerns regarding airflow obstruction, impaired mucociliary clearance and increased susceptibility to infection. Although donor-derived malignancy transmission is rare and primarily documented in case reports, the risk of transmission may increase with advancing donor age and cumulative smoking history.^28^ Generally, studies conclude that outcomes from donors with <20 pack-years (PY) provide similar long-term outcomes compared to non-smoking donors, while findings diverge beyond the 20 PY threshold. Schiavon et al. compared three groups (non-smokers, <20 PY, and ≥20 PY) and found that ≥20 PY was associated with increased intensive care unit (ICU) and hospital stay, while overall survival did not differ.^29^ Using biochemically defined tobacco exposure (e.g. urinary cotinine), Diamond et al. found a moderate increase for PGD 3 at 72 h post-LTx in donor lungs from active smokers, without increasing post-transplant mortality.^30^ In contrast to tobacco smoking, the cumulative effects of vaping and e-cigarettes are not well characterized and the impact on lung quality for organ donation remains unknown. However, in a propensity-matched analysis of 29 LTx from donors with a history of vaping, no differences were observed regarding PGD3, length of hospitalization or pulmonary function tests within the first year after LTx.^31^ Overall, available evidence suggests that carefully selected lungs from smoking donors can be used safely and may meaningfully expand the donor pool. This aligns with the study of Bonser et al, that debated that the life-years potentially lost by accepting lungs from smoking donors were substantially lower than those lost by discarding these organs.^32^ Notably, smoking history is often correlated with other adverse donor features, such as advanced biological age or radiographic abnormalities, complicating causal inference. Functional assessment at procurement, including a collapse test and macroscopic appraisal, may be more informative than smoking history in determining lung suitability.

Furthermore, with the increased legalization of cannabis, ongoing opioid crisis and increasing global drug production and trafficking, substance abuse is increasing and it is estimated that around 316 million people, corresponding to nearly 6% of the population between 15 and 64 years, use drugs.^33^ In a study by Li et al., 39% of all donors accepted for LTx had a history of substance abuse, excluding alcohol consumption, whereas Rappaport et al. reported rates up to 51%.^34^, ^35^ While in the United States, the drug-abuse epidemic has led to an increase in donors dying from overdose (OD), this is not reflected within the Eurotransplant region.^36^ OD donors are usually younger and Xie et al. reported equal outcomes for OD donors compared to other mechanisms of death.^37^ In a UNOS database analysis of 28 495 patients, 12% of patients received lungs from an OD donor.^38^ While recipients from OD donors were intubated longer compared to non-OD, 1- and 3-year survival were equal between both groups.

Although alcohol is a legal and widely consumed psychoactive substance, consumption of excessive amounts of alcohol results in the wear and tear of a donor organ. Previous studies have shown that chronic alcohol abuse induces oxidative stress and depletes antioxidant reserves such as glutathione in the alveolar lining fluid, resulting in impaired epithelial barrier function, altered surfactant homeostasis, and compromised alveolar macrophage function.^39^ Lowery et al. evaluated recipients of lungs from heavy alcohol-use donors and showed that they required longer mechanical ventilation and had an 8-fold higher odds of developing severe PGD compared with recipients of lungs from donors without alcohol abuse.^40^

### PaO₂/FiO₂ ratios < 350 mmHg

Donor oxygenation status as evidenced by the Horowitz index – the ratio of partial pressure of oxygen in arterial blood (PaO₂) to the fractional inspired oxygen (FiO_2_) at 100% – has long been a cornerstone of donor lung assessment. However, oxygenation is a dynamic and context-dependent parameter rather than a direct measurement of the biological quality of the lung. Donor oxygenation can be influenced by several factors including ventilator settings, fluid balance, pulmonary edema, airway secretions and pneumonia, atelectasis, recruitment status, or any other form of reversible lung injury and may therefore not accurately reflect intrinsic graft quality. Atelectasis, particularly involving the lower lobes, is a ubiquitous finding on donor chest computed tomography (CT). In this context, it is essential to distinguish between gravity-related atelectasis and consolidation due to pneumonia, as this differentiation has significant implications for donor suitability. Furthermore, the time between reporting the PaO₂/FiO₂ ratio and procurement might differ, creating a reporting bias in most studies. In an early multicenter study led by Thabut et al, donor oxygenation <350 mmHg was associated with suboptimal gas exchange and decreased long-term outcome in the recipient.^41^ Recently, Zafar et al. showed in a large UNOS registry analysis of 12 000 recipients that donor PaO_2_ before procurement had no association with graft survival, while 20% of all donors had a PaO_2_ ≤200 mmHg.^42^ In our experience, PaO_2_/FiO_2_ was associated with early PGD, however, not with long-term outcomes after LTx.^43^ Conversely, higher donor PaO_2_ has been shown to be associated with the development of bronchiolitis obliterans syndrome after LTx.^44^ Overtly high PaO₂/FiO₂ ratios can indicate air-trapping caused by emphysema.

These findings challenge the reliance on rigid oxygenation thresholds of traditionally >350 mmHg and support a more nuanced approach incorporating dynamic assessment and contextual interpretation. This evidence suggests that a low PaO₂/FiO₂ ratio should therefore not lead to upfront donor decline when the clinical context suggests potentially reversible hypoxemia. Instead, low oxygenation should prompt active donor optimization, including bronchoscopy, airway clearance, lung recruitment maneuvers, adjustment of ventilator settings, evaluation of fluid balance, and reassessment after intervention. During procurement, visual inspection and guided recruitment maneuvers can drastically increase PaO₂/FiO₂. If peripheral PaO₂/FiO₂ remains low, direct sampling from the right and left pulmonary veins can give an indication of the quality of each lung. Aziz et al. showed that pulmonary veins analysis can identify transplantable lungs despite low peripheral PaO₂, with comparable early graft function and survival in recipients receiving lungs with peripheral PaO₂ <300 mmHg but pulmonary veins PO₂ >300 mmHg.^45^ If there a huge mismatch between one side and the other, donation for single LTx can be considered.

### X-ray abnormalities and trauma

Although the lungs are protected by the thoracic cage, both trauma and cardiopulmonary resuscitation efforts can result in aspiration of gastric contents, pulmonary injury, and hence aberrant radiology. In a single-center report published in 2010, approximately 12% of donor lung offers were declined based solely on imaging findings.^46^ Common abnormalities identified after trauma include pneumothorax, hemothorax, and pulmonary contusion. While pneumothorax and hemothorax can often be effectively managed with chest tube drainage, pulmonary contusion requires more careful assessment and may necessitate further evaluation using EVLP. However, successful LTx has been reported in donors with pulmonary contusion, indicating that such findings are not absolute contraindications for LTx.^47^ Radiographic abnormalities in donor lungs can be highly variable, and interpretation is often limited by interobserver variability and subjectivity. Consequently, findings on chest radiography alone are generally not considered definitive contraindications to transplantation.

However, with the increasing availability and utilization of CT, donor lung evaluation is progressively shifting towards thoracoabdominal CT as a more comprehensive imaging modality.^48^ This is particularly relevant in ECD in whom CT imaging can help identify abnormalities that can’t be identified on chest x-rays such as pulmonary embolisms. Notably, pulmonary embolism as a cause of death is not considered an absolute contraindication to LTx. In a study from Sommer et al, LTx from donors deceased due to pulmonary embolism resulted in equal PGD rates and long-term survival compared to donors without pulmonary embolism.^49^ Although the impact of donor pulmonary hypertension (PHT) on post-LTx outcome is not well characterized, it often serves as a relative contraindication. Only recently, Jafari et al. showed that lung utilization was lower in donors with PHT, although short- and long-term survival was comparable to donors without PHT.^50^ In addition, the possibility of fat embolism, particularly in trauma donors with long bone fractures such as the femur, should be considered.^51^ Although significant clots can be clearly recognized during retrograde flush, the clinical relevance of fat embolism in donor lung assessment remains less well defined, as highlighted by two reported cases of donor-acquired fat embolism syndrome, mimicking severe PGD.^52^ In one case, CT findings showed consolidations in the allograft after unilateral LTx, while there was no evidence for macroscopic obstruction and transesophageal echocardiogram demonstrated no impairment of the arterial and venous anastomoses. Only histological analysis of the non-transplanted lung revealed the presence of adipose island in the pulmonary arterioles. Nevertheless, caution remains essential when interpreting imaging findings, and radiological assessment should always be integrated with clinical, functional, and intraoperative evaluation.

### Microbial colonization

Pulmonary infections remain a major source of morbidity and mortality after LTx. Lungs are continuously exposed to the external environment making them more prone to infections and are susceptible to ventilator-associated injury and nosocomial infection during intensive care management. Aspiration events, which are common in organ donors, further predispose the lung to microbial injury by facilitating the entry of oropharyngeal and gastric flora into the lower respiratory tract. Microbial colonization in donor lungs can be assessed using bronchoalveolar lavage (BAL), bronchial aspirates or swabs, tissue sampling, or preservation fluid analysis, which may result in variable microbiological yield across sampling techniques. While BAL positivity of donors mostly ranges from 36% to 46%, Bonde et al. reported that swabs of the opened bronchus were positive in 89% of cases, mostly comprising Gram-positive cocci.^53^, ^54^ Analysis of preservation solution showed positivity in one third of cases with frequently isolated organisms including Staphylococcus aureus and Escherichia coli.^55^ While donor-derived microorganisms have been associated with an increased early morbidity, including prolonged mechanical ventilation and ICU stay, causality between donor lung colonization and post-transplant pneumonia and long-term outcomes remains unclear.^56^

Interestingly, clinical characteristics appear to have limited predictive value for microbiological findings. Fumagalli et al. demonstrated that standard donor-related variables such as donor type and Oto-score do not predict BAL positivity.^53^ Consequently, the specific impact of ECD on microbial positivity remains insufficiently characterized. Taken together, these findings indicate that microbial positivity in donor lungs is common and should not be considered an absolute contraindication to transplantation.

### Donation after circulatory death

DCD has emerged as one of the most impactful strategies of the 21st century for expanding the donor pool.^6^ Unlike DBD donors, DCD donors experience a period of warm ischemia following withdrawal of life-sustaining therapy, initially raising concerns regarding ischemic injury. However, compared to other solid organs, the lung is privileged by the residual alveolar oxygen reserves during the asystolic phase and during preservation. Furthermore, brain death triggers a systemic inflammatory response in which catecholamine-driven hemodynamic injury and elevated cytokines (IL-6, IL-8, TNF-alpha, etc.) increase pulmonary permeability.^57^ Over time, skepticism regarding DCD has gradually given way to broader acceptance as experience has accumulated. Just like ECD, advances in donor management, procurement techniques, and preservation strategies have been instrumental in mitigating this ischemic injury in DCD.

Multiple single- and multicenter studies have now demonstrated that clinical outcomes following controlled DCD are comparable to those after DBD. In an ISHLT registry analysis based on data from selected high-volume lung transplant centers in North-America, Europe, and Australia, Van Raemdonck et al. demonstrated comparable long-term survival between DBD and DCD.^58^ In 2025, Spadaccio et al. extended these findings in a meta-analysis, showing comparable rates of PGD between DCD and DBD, alongside a modest but statistically significant improvement in five-year survival with DCD.^59^ Some studies have reported a higher incidence of airway complications following transplantation with DCD lungs compared with DBD, although these findings remain inconsistent and are likely influenced by confounding donor and recipient characteristics rather than donor type alone.^60^ However, in a recent European multicenter analysis comprising more than 5000 patients, it was demonstrated that DCD should not be considered a risk factor for airway complications.^61^

The impact of DCD heart transplantation and uncontrolled donation on long-term outcome after LTx remain incompletely understood. In this context, (thoraco)abdominal normothermic regional perfusion introduces additional complexity to donor selection and procurement. Additionally, patients who are already on cardiopulmonary support including ECMO or Impella in the ICU, are becoming increasingly considered for organ donation.^62^ Compared to direct procurement, these new strategies are associated with longer donor warm ischemia time for the lungs. Although early experience suggests that these strategies are feasible, their specific impact on lung graft quality and post-transplant outcomes has yet to be clearly defined and larger prospective studies are awaited.^63^ While these strategies aim to expand the overall donor pool, it is imperative to safeguard lung utilization, ensuring that the pursuit of quantity does not compromise quality.

### Prolonged ischemic time and controlled hypothermic storage

Traditionally, static preservation on ice has been the standard in lung storage for decades. Although initially thought that ice stored the organ at approximately 4°C, recent evidence showed that the actual temperature approximates 0°C, proning the lung to freezing injury with an associated increased risk for PGD.^64^ Furthermore, despite the longstanding paradigm that shorter ischemic times were associated with superior transplant outcomes, improvements in preservation techniques have challenged this concept. Although EVLP was initially used to extend ischemia time of the lung, for example in combined liver-lung transplantation (liver-first strategy), controlled hypothermic storage has drastically improved the preservation options for the lung.^65^ These devices maintain a stable temperature environment by preventing freezing injury and reduce ischemia-reperfusion injury, thereby preserving graft quality during prolonged preservation. These innovations have facilitated safe extension of ischemic times beyond traditional limits of more than 6 h.

Ali et al. described their first experience with storage using 10°C preservation, illustrating no difference in short-term outcomes including PGD and postoperative ECMO support.^66^ In a first European multicenter analysis, Provoost et al. demonstrated excellent postoperative outcomes for lungs preserved in a controlled hypothermic cooling system, while Novysedlak et al. showed the feasibility of preservation >15 h.^67^, ^68^ Hoetzenecker et al. recently demonstrated that even prolonged preservation exceeding 24 h results in excellent postoperative outcomes without impact on short-term patient survival.^69^

The ability to reliably extend preservation times using controlled hypothermic storage has enabled a paradigm shift toward semi-elective transplantation strategies. This strategy allows alignment with daytime operating conditions, which may reduce fatigue and support optimal team performance, even though outcomes between daytime and nighttime transplantation appear comparable in experienced centers.^70^ Beyond procedural optimization, prolonged preservation expands the donor pool by enabling long-distance procurement, facilitating immunological matching, and providing logistical flexibility for complex or overlapping transplant scenarios.

## Emerging extended-criteria donors

Despite the steady increase in organ availability afforded by ECD, organ shortage continues to constrain organ transplantation. In response, donor comorbidities that would previously have been considered an absolute contraindication have increasingly been accepted in recent years. In contrast to typical ECD, these represent standalone donor characteristics that intrinsically carry a higher risk, which must be carefully balanced against recipient characteristics and sometimes require additional post-transplant medical management.

### Hepatitis C virus–positive donors

Donors with hepatitis C virus (HCV) represent a growing pool, comprising mostly young donors who died from drug overdose. A landmark study demonstrated that 36 HCV-viremic donor lungs were safely transplanted into HCV-naïve recipients, effectively preventing chronic HCV transmission when direct-acting antiviral therapy was initiated immediately after transplantation.^71^ Building on this evidence, the 2020 ISHLT expert consensus statement formalized both prophylactic and preemptive treatment strategies for the use of HCV-viremic donor organs.^72^

### Donors with SARS-CoV-2 infection

During the COVID-19 pandemic, transplantation using lungs from SARS-CoV-2 PCR-positive donors was reported successfully despite initial reluctance.^73^, ^74^ A larger UNOS analysis demonstrated no difference in early mortality or graft failure among 51 recipients of lungs from SARS-CoV-2-positive donors compared to negative donors.^75^ However, a more recent single-center analysis spanning the Omicron era showed that 11 recipients of lungs from SARS-CoV-2-positive donors had a significantly higher 30-day mortality (18.2% *versus* 3.6% for negative donors), although 1-year survival did not differ.^76^ Opposed to nasal swab positivity, donors with a positive lower respiratory tract test carry a higher risk, resulting in potential post-transplant infection. Current guidelines vary between countries but generally require a negative donor PCR. When an initial test is positive, repeat testing can help distinguish resolving from active infection, and growing evidence supports selective acceptance of SARS-CoV-2-positive donors, particularly when lower respiratory tract sampling is negative.

### Donors with a history of asthma

Another clinical challenge remains a donor history of asthma or even status asthmaticus as the cause of death. In 2004, Oto et al reported no significant difference in survival nor pulmonary function testing for recipients of asthmatic donor lungs compared with non-asthmatic lungs.^77^ For asthmatic lungs, the authors distinguish between donors on treatment (N=22) and without treatment (N=13) for their asthma. Although non-significant, the mortality at 30 days was higher for the no-treatment group (23%) compared to the treatment group (9%). Ultimately, the authors recommend caution with fatal asthmatic donors, given both recipient in their cohort suffered from graft dysfunction and died after 73 and 484 days, respectively. A recent case report by Park et al. describes severe asthma exacerbations requiring post-transplant treatment in a non-asthmatic recipient who received lungs from an asthmatic donor.^78^ Consequently, carefulness is warranted especially for non-treated asthmatic donors, requiring a critical macroscopic appraisal and collapse test indicative of active bronchospasm or exacerbation at the time of procurement.

### Donors with a history of malignancy

Finally, donors with a history of a malignancy constitute an increasingly recognized category, historically underutilized because of concerns about donor-derived transmission. Consensus frameworks stratify donor malignancies into risk categories, from minimal to high, based on tumor type, grade, stage, and disease-free interval, providing a structured basis for donor acceptance decisions.^79^ A frequently cited example is the incidental finding of a renal cell carcinoma during donor work-up, where a small (≤1 cm), well-differentiated (Fuhrman 1–2) tumor is generally considered a minimal-risk finding that does not preclude organ use. A recent UNOS registry study illustrates the extent of underutilization in LTx specifically: of 935 potential donors with a cancer diagnosis at procurement, only 13% ultimately had their lungs transplanted, with intracranial tumors utilized more frequently than extracranial malignancies (25% vs 7.6%).^80^ Survival did not differ between recipients receiving lungs from donors with and without a cancer history, and donor-derived transmission was <1% in both groups. Recently, Greenhall et al, reported no donor-derived transmission in 778 transplants from 282 multi-organ donors with primary brain tumors.^81^ Nevertheless, lung utilization was only 16%, reflecting continued reluctance among LTx centers to accept these donors.

## The role of ex vivo lung perfusion

Since its clinical introduction in 2000, EVLP has significantly advanced the field of LTx by enabling normothermic functional assessment and reconditioning of donor lungs outside the body. By maintaining organs under near-physiological conditions, EVLP allows real-time evaluation of gas exchange, pulmonary vascular resistance, compliance, and inflammatory profiles. Clinical trials have demonstrated that lungs falling outside conventional criteria can be successfully transplanted following EVLP assessment and optimization.^82^, ^83^ However, with increasing experience in ECD utilization and the emerging controlled hypothermic storage devices, EVLP is no longer universally regarded as the standalone preservation method.

Nevertheless, in some countries EVLP continues to play an important role in the assessment of borderline quality donor lungs, while EVLP is still compulsory for the evaluation of DCD lungs in France. In this context, EVLP can be especially useful for lungs with a persistently low PaO₂/FiO₂ ratio despite optimal management as well as DCD lungs with prolonged warm ischemic times, such as in DCD heart procurement or uncontrolled donation. Furthermore, beyond its current role, EVLP may evolve into a therapeutic platform for advanced organ repair and engineering. In preclinical models using discarded human donor lungs, EVLP with high-dose antibiotic treatment has shown promising results with reductions in bacterial load and improvements in graft function.^84^ In addition, strategies aimed at modulating the inflammatory milieu, may further enhance graft quality and early post-transplant outcomes. In a pilot study by Cypel et al, airway delivery of an adenoviral vector encoding human interleukin-10, was shown to improve pulmonary function after EVLP.^85^ Looking ahead, generating more universally acceptable lungs by silencing MHC expression or converting blood group antigens, as demonstrated in preclinical models by the Toronto group, underscores the transformative potential of EVLP beyond classical organ assessment.^86^, ^87^

## Conclusions

The expanding use of ECD lungs reflects both necessity and innovation in modern LTx. Accumulating evidence supports the safe utilization of lungs previously considered marginal, particularly when combined with advanced assessment and preservation technologies. Despite these encouraging data, the evidence supporting extended-criteria lung utilization is subject to important limitations. Most studies are retrospective and observational, with inherent selection bias and residual confounding. High-volume centers with advanced resources may achieve outcomes that are not reproducible elsewhere. Additionally, existing risk stratification tools inadequately capture the complex interplay between donor, recipient, and procedural factors. While advances in molecular profiling and artificial intelligence may further refine donor lung assessment in the future, current decision-making remains primarily guided by the interpretation of physiological, radiological and microbiological parameters. Moving toward a more individualized, data-driven evaluation, thereby balancing donor risks against recipient profiles and center expertise, is essential to sustainably expand the donor pool. By redefining acceptable donor criteria we will not eliminate organ shortage, but it represents a critical step toward reducing waitlist mortality and improving access to life-saving LTx.

## Disclosures

None.

## Declaration of Generative AI and AI-assisted technologies in the writing process

Artificial intelligence and assisted tools were used to support language editing and stylistic refinement of the original manuscript. These tools were not used for data generation, data analysis, interpretation of results, or any aspect of the original research.

## Declaration of Competing Interest

The authors declare that they have no known competing financial interests or personal relationships that could have appeared to influence the work reported in this paper.
